# Antimicrobial-loaded PMMA bone cement: time for good practice recommendations

**DOI:** 10.1093/jac/dkag261

**Published:** 2026-08-20

**Authors:** Tariq Azamgarhi, Herbert Gbejuade, Ryan A Hamilton, Simon Warren, Elizabeth S R Darley, Mark Gilchrist, Erden Ali, Félix–Antoine Garneau–Picard, Jason C J Webb

**Affiliations:** Pharmacy Department, Royal National Orthopaedic Hospital NHS Trust, Stanmore, UK; Bone Infection Unit, Royal National Orthopaedic Hospital NHS Trust, Stanmore, UK; Department of Orthopaedic Surgery, Southmead Hospital, Southmead Road, Westbury on Trym, Bristol BS10 5NB, UK; Faculty of Health and Life Sciences, Leicester School of Pharmacy, De Montfort University, Hawthorn Building, The Newarke, Leicester LE2 7BY, UK; Bone Infection Unit, Royal National Orthopaedic Hospital NHS Trust, Stanmore, UK; Department of Medical Microbiology, Southmead Hospital, Southmead Road, Westbury-on-Trym, Bristol BS10 5NB, UK; Department of Pharmacy, Imperial College Healthcare NHS Trust, Charing Cross Hospital, Fulham Palace Road, London W6 8RF, UK; Joint Reconstruction Unit, Royal National Orthopaedic Hospital NHS Trust, Stanmore, UK; Bone Infection Unit, Royal National Orthopaedic Hospital NHS Trust, Stanmore, UK; Division of Infectious Diseases and Medical Microbiology, CHU de Québec–Université Laval, Québec, Québec, Canada; Department of Orthopaedic Surgery, Southmead Hospital, Southmead Road, Westbury on Trym, Bristol BS10 5NB, UK

## Abstract

Antimicrobial-loaded PMMA bone cement has been used since the 1970s as both a fixation material and a local antimicrobial reservoir. Its clinical roles differ between prophylaxis in primary arthroplasty and treatment of established infection, particularly during revision surgery and through the use of temporary spacers. Commercially manufactured products offer more predictable handling, but bespoke admixing may be considered when resistant or unusual organisms are encountered. This Viewpoint argues that such practice requires clearer governance. Cement formulation, antimicrobial choice, dose and mixing technique influence elution, bioactivity, mechanical strength and toxicity. Local delivery may support antimicrobial stewardship, but prolonged low-level elution could theoretically select for resistance if poorly planned. We propose a cautious, evidence-informed framework to support multidisciplinary decision-making and the development of PMMA-specific antimicrobial guidance.

## Introduction

Periprosthetic joint infection (PJI) remains one of the most serious complications of joint arthroplasty and accounts for a substantial proportion of revision procedures recorded in national joint registries.^1,2^

The concept of antimicrobial-loaded polymethyl methacrylate (PMMA) bone cement was pioneered in the 1970s when Buchholz and Engelbrecht incorporated gentamicin into Palacos cement to achieve a local depot effect.^3^ Since then, PMMA has become an important adjunct in orthopaedic practice because it can combine structural function with local antimicrobial release.

Its clinical use should not be considered a single entity. In primary arthroplasty, antimicrobial-loaded cement is generally used prophylactically, usually as a commercially manufactured low-dose product according to local policy and patient risk. In revision surgery or established infection, particularly in two-stage exchange, spacers and fixation constructs may require higher doses or microbiology-directed combinations, with different implications for elution, mechanical safety, toxicity and stewardship.

## Evolving evidence for local antimicrobial delivery

The effectiveness of local antimicrobial delivery as part of operative infection management has been recognized for several decades. Selected series of two-stage revision arthroplasty have reported successful outcomes despite short postoperative systemic courses, provided that surgical source control and local antimicrobial delivery are achieved.^4^

The SOLARIO protocol has brought this stewardship question into sharper focus. SOLARIO was designed as a multicentre, open-label, randomized, non-inferiority trial comparing short systemic antimicrobial therapy of 7 days or less with standard systemic therapy of 4 weeks or more in adults with orthopaedic infection treated operatively with local antimicrobial therapy. The planned sample size was 500 participants, with treatment failure at 12 months as the primary outcome and a prespecified non-inferiority framework.^5^ Full peer-reviewed outcome data should be awaited before drawing definitive conclusions, but the protocol illustrates the central clinical question: whether local antimicrobial strategies can safely reduce systemic exposure when combined with definitive surgery and source control.

Several local carriers have been explored, including calcium sulphate, ceramics and hydrogels. PMMA remains distinctive because it can act both as a fixation material and as a local antimicrobial reservoir. This dual role is valuable, but it also creates risk: adding antimicrobials can alter setting, porosity, elution and mechanical performance.^6–10^

Commercially manufactured antimicrobial-loaded PMMA products have better characterized handling and performance than *ad hoc* manual admixture. Nonetheless, antimicrobial resistance, unusual organisms and allergy may require bespoke local combinations. This is where practice becomes most variable and where good-practice guidance is most needed.

## Why guidance is needed

Current practice around antimicrobial loading of PMMA varies among centres, surgeons, infection teams and countries. Variation is partly unavoidable because microbiology, product availability, licensing and local resistance patterns differ internationally. It becomes problematic when drug choice, dose, cement type and mixing method are selected intra-operatively without a documented rationale.

This Viewpoint therefore proposes a good practice framework, rather than a formal guideline or practice standard (Table 1). The framework is intended to support multidisciplinary decision-making in areas where formal guidance remains limited and should be interpreted alongside local policy, product licensing, microbiology advice and national recommendations.

**Table 1.** Proposed good practice framework for antimicrobial-loaded PMMA

Workflow	Good practice consideration	Rationale
**Deliberation (pre-operative planning)**	Define the indication and microbiological intent before cement selection or admixture.	Separates prophylaxis in primary arthroplasty from therapeutic use in revision or established infection, and supports targeted selection.
	Apply antimicrobial stewardship principles to local as well as systemic therapy.	Local delivery should be justified by drug, dose and delivery method, rather than regarded as automatically beneficial.
	Plan, prescribe and supply antimicrobials pre-operatively where possible.	Reduces intra-operative delay, preparation errors and unplanned broad-spectrum admixture.
**Drug (antimicrobial selection)**	Use commercially manufactured antimicrobial-loaded PMMA where suitable and available.	Provides better-characterized handling, dosing, elution and mechanical performance than unstandardized manual admixture.
	When bespoke local therapy is required, select agents according to microbiology, allergy, renal risk, local resistance patterns, licensing and local practice.	Recognizes international variation, including different aminoglycoside preferences and avoids unnecessary broad-spectrum combinations.
	Use PMMA-specific antimicrobial guidance where available.	Drug-specific cement guidance should address formulation, dose per 40 g of cement, thermal stability, mixing, elution, mechanical effects and toxicity.
	Prefer powder preparations over liquids when admixing.	Liquid preparations may impair polymerization and reduce cement strength.^6^
	Avoid agents with poor thermal stability, poor elution or adverse effects on polymerization.	Some antimicrobials are inactivated by curing conditions or compromise setting and mechanical properties.^11,12^
**Dosing (mechanical safety)**	Use conservative doses when PMMA is used for fixation or expected to provide structural support.	Helps preserve mechanical properties and reduce the risk of systemic toxicity.^7,13^
**Delivery (carrier, cement and technique)**	Select cement type according to the intended balance between elution and mechanical demand.	Palacos-type cements generally show higher elution than Simplex-type cements, but structural requirements remain important.^8^
	Standardise and document the mixing technique and the quantities of antimicrobial and cement used.	Improves reproducibility; porosity, powder-liquid ratio and technique influence elution and strength.^14^
**Delivery (alternative carriers)**	Consider alternative local carriers where PMMA is unsuitable for the selected antimicrobial or clinical objective.	Calcium sulphate, ceramics and other carriers may offer different release characteristics, but they do not provide PMMA’s fixation function.

This framework is intended to support multidisciplinary decision-making. It is not a formal guideline and should be applied alongside local policy, product licensing, microbiology advice and national recommendations.

Antimicrobial stewardship is central to this discussion. Local antimicrobial delivery may reduce unnecessary systemic exposure in selected settings, but this does not necessarily translate into a stewardship benefit. Prolonged low-level elution may create sub-inhibitory local concentrations, with a theoretical risk of resistance selection, particularly if broad-spectrum combinations are used without microbiological justification.^9^ The stewardship principles applied to systemic therapy should therefore also apply locally: define the indication, choose the narrowest-spectrum effective agent, justify the dose and document the intended local antimicrobial strategy.

The term PMMA-specific antimicrobial guidance is used here deliberately. We are not referring to standard drug monographs. Rather, we suggest drug-specific cement guidance covering formulation, powder versus liquid preparation, thermal stability, dose ranges per 40 g of cement, mixing method, elution behaviour, mechanical effects, toxicity risks and relevant evidence.

## Mechanical effects of antimicrobial addition

PMMA may be load-bearing, and weakening it can have serious clinical consequences. The effect of antimicrobial addition is dose-dependent, cement-specific and drug-specific. In vitro work has shown that vancomycin loading and cement formulation influence compressive strength and elution, with some combinations showing progressive strength reduction after elution ageing.^6^

Mechanical performance may also deteriorate between stages of a two-stage revision. Dual-antibiotic PMMA spacers can lose strength over six weeks, emphasizing the need to consider spacer design, protected weight-bearing, cement choice and the mechanical demands placed on the construct.^7^

Cement selection matters. Comparative studies have shown that Palacos-type cements generally elute more antibiotic than Simplex-type cements across several agents, although higher elution does not remove the need to preserve adequate mechanical properties for the intended clinical role.^8^

The same caution applies to emerging combinations. Published *in vitro* daptomycin-PMMA data support antimicrobial effectiveness, elution and acceptable mechanical stability at clinically relevant doses.^15^ These findings are encouraging, but they remain laboratory data and should not be treated as clinical proof of safety or efficacy for all daptomycin-containing combinations.

## Optimising elution and bioactivity

Only a proportion of the antimicrobial incorporated into PMMA elutes in a bioactive form. Elution is passive and surface-driven, typically with an early burst over the first 24–48 h followed by a prolonged tail over subsequent weeks. A substantial proportion of the antimicrobial remains trapped within the cement matrix.^10^

Drug choice and combinations influence elution. Gentamicin is a consistently efficient elutor from PMMA, and aminoglycoside-containing combinations can improve elution of partner drugs compared with single-agent loading.^10^ Such findings are useful for formulation decisions, but they should be interpreted alongside microbiology, toxicity and mechanical requirements rather than used to justify broad empirical loading.

Technique also matters. Vacuum mixing, fractionated mixing, powder-liquid ratio, porosity and hydrophilic copolymers can all influence elution and reproducibility. Methods that increase porosity may improve release but can also increase early exposure and weaken the cement matrix.^14^ This trade-off explains why elution should not be optimized in isolation.

## Systemic and local toxicity

Local antimicrobial delivery aims to achieve high local concentrations while limiting systemic exposure, but measurable serum levels and systemic toxicity can occur. Reports are difficult to interpret because surgery, infection severity and concurrent systemic antimicrobials are important confounders. Nonetheless, systematic reviews and cohort studies show that acute kidney injury may complicate high-dose antimicrobial spacer use, particularly in frail patients, those with pre-existing renal impairment and those receiving concurrent nephrotoxic agents.^13,16,17^

Variability in elution and systemic absorption between cement brands and mixing methods makes risk difficult to predict without standardized dosing guidance. Local tissue toxicity at the bone-cement interface has also been demonstrated experimentally, although the clinical significance requires further definition.^18^

## Drug-specific risks and practical constraints

Not all antimicrobials are suitable for incorporation into PMMA. Heat-labile agents may degrade during cement polymerization, while some drugs interfere with polymerization or setting. Liquid preparations can alter the powder-liquid ratio and weaken the cement matrix, and some agents show poor elution despite *in vitro* antimicrobial activity.^6,11,12^

These constraints are one reason why local practice cannot be reduced to a single preferred antimicrobial. Gentamicin is historically important and widely used, but tobramycin may be preferred or more readily available in some countries. Vancomycin should not be treated as a routine addition without microbiological or patient-specific justification. Drug choice should reflect the clinical indication, culture results, allergy, renal risk, local resistance patterns, licensing and product availability.

### Conclusion

Antimicrobial-loaded PMMA remains an important adjunct in arthroplasty and orthopaedic infection management because it can combine structural function with local antimicrobial delivery. Its value, however, depends on using it safely, reproducibly and with appropriate stewardship.

The current evidence supports caution rather than universal prescribing. Cement type, antimicrobial choice, dose and mixing method all influence elution, bioactivity, mechanical strength and toxicity. Local delivery may help reduce systemic exposure in selected patients, but may also create prolonged low-level antimicrobial release if poorly planned. Good practice therefore requires a clear indication, microbiology-guided selection, conservative dosing when PMMA is load-bearing, standardized mixing and documentation. PMMA-specific antimicrobial guidance would provide a practical next step for surgeons, infection physicians, pharmacists and theatre teams.
